# Long noncoding RNA IRF1-AS is associated with peste des petits ruminants infection

**DOI:** 10.1186/s13567-022-01105-1

**Published:** 2022-10-28

**Authors:** Bo Wen, Xuefeng Qi, Daiyue Lv, Lulu Yang, Pan Tang, Wenchi Chang, Shuizhong Han, Shengmeng Yu, Shaopeng Wei, Qinghong Xue, Jingyu Wang

**Affiliations:** 1 College of Veterinary Medicine, Northwest A&F University, Yangling, 712100 Shaanxi China; 2 China Institute of Veterinary Drug Control, Beijing, 100000 China

**Keywords:** PPRV, lncRNAs, innate immune response, IRF1, IRF3

## Abstract

Peste des petits ruminants (PPR) is an acute and highly contagious disease and has long been a significant threat to small ruminant productivity worldwide. However, the molecular mechanism underlying host-PPRV interactions remains unclear and the long noncoding RNAs (lncRNAs) regulation of PPR virus (PPRV) infection has rarely been reported so far. Here, we first demonstrated that PPRV infection can induce an obvious innate immune response in caprine endometrial epithelial cells (EECs) at 48 h post-infection (hpi) with an MOI of 3. Subsequently, we determined that PPRV infection is associated with 191 significantly differentially expressed (SDE) lncRNAs, namely, 137 upregulated and 54 downregulated lncRNAs, in caprine EECs compared with mock control cells at 48 hpi by using deep sequencing technology. Importantly, bioinformatics preliminarily analyses revealed that these DE lncRNAs were closely related to the immune response. Furthermore, we identified a system of lncRNAs related to the immune response and focused on the role of lncRNA 10636385 (IRF1-AS) in regulating the innate immune response. Interestingly, we found that IRF1-AS was a potent positive regulator of IFN-β and ISG production, which can significantly inhibit PPRV replication in host cells. In addition, our data revealed that IRF1-AS was positively correlated with its potential target gene, IRF1, which enhanced the activation of IRF3 and the expression of ISGs and interacted with IRF3. This study suggests that IRF1-AS could be a new host factor target for developing antiviral therapies against PPRV infection.

**Supplementary Information:**

The online version contains supplementary material available at 10.1186/s13567-022-01105-1.

## Introduction

Peste des petits ruminants virus (PPRV), the causative agent of peste des petits ruminants (PPR) disease, has a linear negative-stranded RNA genome and belongs to the genus *Morbillivirus* in the family *Paramyxoviridae* [1]. PPR is an acute, highly contagious fatal disease that mainly affects goats and sheep, although it also occasionally affects small or even large wild ruminants [2–4]. Currently, PPRV has spread to many countries, and approximately 80% of goats and sheep in the world are threatened by this virus, which causes significant economic losses [5–7]. The disease is clinically characterized by a high fever, mucopurulent oculonasal discharges, diarrhoea, stomatitis and pneumonia symptoms [8, 9]. In addition, PPRV infection often causes foetal mummification and abortion [10, 11]. Live attenuated vaccines have been used to control PPR and have shown a good immunological effect on both sheep and goats [12, 13]. Among these live attenuated vaccines, the Nigeria/75 (N75) vaccine has been shown in different studies to protect against viral isolates of all 4 lineages in most countries [13, 14]. Like all morbilliviruses, PPRV has a well-established lymphatic and epithelial tissue tropism [15, 16]. Therefore, caprine endometrial epithelial cells (EECs) are used as standard in vitro models to study host-PPRV interactions [17–19]. Recently, transcriptome analysis revealed transcription factors modulating immune responses in PPRV-infected host cells [20, 21]. However, our understanding of the role of cellular lncRNAs in EECs,—a cell line of the reproductive system—, during PPRV infection is unknown.

lncRNAs are a large class of noncoding RNAs that are more than 200 bp in length and have no or limited coding potential [22, 23]. lncRNAs regulate gene expression by regulating transcription factors, inducing chromatin modification, affecting RNA processing events, sponging microRNAs (miRNAs), and affecting RNA stability [24]. lncRNAs are involved in many biological processes, including immunity and inflammation [25, 26]. Notably, lncRNAs participate in the battle between host and virus via the transcription of viral and host genes, stability and translation of mRNAs, and host antiviral responses [27]. For instance, the lncRNA Lnczc3h7a promotes a TRIM25-mediated RIG-I antiviral innate immune response [28]; Lnc-ISG20 inhibits influenza A virus replication by enhancing ISG20 expression [29]; the lncRNA AVAN promotes antiviral innate immunity by interacting with TRIM25 and enhancing the transcription of FOXO3a [30]; and the lncRNA NRAV can regulate the replication of influenza A virus through inhibition of interferon-stimulating genes such as IFITM3 and MxA [31]. In addition, a viral infection can trigger changes in the cellular lncRNA profile, which can greatly influence the pathogenesis of viral diseases [32–34].

Although the innate immune system is the first line of defence against microbial invasion, many viruses have developed strategies to evade and antagonize the host immune response and resist the antiviral actions of IFN therapy [35]. PPRV can also cause immunosuppression in natural hosts, which benefits viral replication in infected cells [6, 36]. Recently, studies have indicated that PPRV N protein inhibits IFN-β production and signalling by interacting with IRF3 to block its activation [37]; PPRV-induced novel miR-3 contributes to inhibiting type I IFN production by targeting IRAK1 [38]. However, how the host regulates cellular responses to alleviate innate immunosuppression and enhance the innate immune response to restrict viral replication is relatively poorly studied. Our previous study revealed that FANCL, a host protein, induced type I IFN production by promoting TBK1 phosphorylation, thus impairing PPRV-mediated immunosuppression and inhibiting PPRV replication [39]. However, whether lncRNAs also participate in the process of enhancing the innate immune system to counteract this immunosuppression is unknown. Therefore, in the present study, we hypothesized that PPRV could regulate the expression of lncRNAs in infected EECs and that lncRNAs exist as host cell factors to counteract innate immunosuppression and suppress viral replication. To test this hypothesis, we performed next-generation sequencing to identify differentially expressed (DE) lncRNAs in caprine EECs infected with PPRV. We found that PPRV infection deeply changed the lncRNA expression profile. Gene Ontology (GO) and Kyoto Encyclopedia of Genes and Genomes (KEGG) pathway enrichment analyses of significantly differentially expressed (SDE) lncRNAs predicted their putative regulatory roles in the antiviral response to PPRV infection. Moreover, we identified lncRNA 10,636,385 (IRF1-AS), which is critical for the regulation of innate immune responses and the inhibition of PPRV replication. And IRF1-AS enhanced type I IFN production and ISG expression by regulating interferon regulatory factor 1 (IRF1) expression which promoted the activation of IRF3. To the best of our knowledge, this is the first study to reveal the lncRNA expression profile of goat reproductive system cells in response to PPRV infection. lncRNAs can play an antiviral function against PPRV by enhancing the host innate immune response and therefore impairing PPRV-mediated immunosuppression.

## Materials and methods

### Cells, viruses and antibodies

Caprine EECs were immortalized by transfection with human telomerase reverse transcriptase (hTERT), and we have previously confirmed that the secretory function of these cells is consistent with that of primary EECs [40, 41]. The cells were kindly provided by Prof. Yaping Jin (Northwest A&F University Yangling, Shaanxi, China). EECs and primary GFF cells were cultured in Dulbecco’s modified Eagle medium/F-12 Ham’s medium (DMEM/F12; Gibco, Carlsbad, CA, USA) supplemented with 10% foetal bovine serum (Gibco), 100 IU/mL penicillin, and 10 µg/mL streptomycin (Gibco) at 37 °C in 5% CO_2_. The PPRV attenuated strain Nigeria 75/1 was obtained from our laboratory culture collection. The viral stock was prepared by collecting infected cell supernatant when a cytopathic effect (CPE) was apparent in approximately 80% of the cells. To determine the viral titres (50% endpoints), cells cultivated in 96-well plates were inoculated with 10-fold serial dilutions of the virus and incubated at 37 °C for 5–7 days.

Anti-PPRV-N monoclonal antibody was provided by the China Animal Health and Epidemiology Centre (Qingdao, China). Specific antibodies against IRF1 and IRF3 were purchased from Santa Cruz Biotechnology (Santa Cruz, CA, USA). Anti-anti-p-IRF3 antibody was purchased from Abcam (Cambridge, MA, USA). Anti-β-actin antibody, HRP-conjugated secondary antibodies, Transgen Biotechnology (Beijing, China). TRITC-phalloidin was purchased from Sigma.

### RNA isolation and real-time PCR analysis

TRizol reagent was used to extract the total RNA of goat cells according to the manufacturer’s instructions (Invitrogen, Waltham, MA, USA). Reverse transcription was carried out using M-MLV reverse transcriptase (Invitrogen, Waltham, MA, USA). qRT-PCR was performed using SYBR Green master mix (TransGen Biotech, China) to quantify the RNA copy numbers on an iQ5 qRT-PCR System (Bio-Rad, USA). The PCR cycling conditions were 2 min at 95 °C followed by 40 cycles of 15 s at 94 °C and 30 s at 60 °C. Relative abundance of LncRNA and mRNA transcripts were analysed and calculated by the threshold cycle method (2^−ΔΔCt^). The relative expression level of each gene was normalized to housekeeping gene β-actin. All specific primers used in this study are listed in Table 1.

**Table 1:** **qRT-PCR primers used in this study**

Target gene	Forward primer (5′-3′)	Reverse primers (5′-3′)
β-actin	CACGGTGCCCATCTACGA	CTTGATGTCACGGACGATTT
IRF1-AS	GCAGCCCAGGACCAGACTT	TGATAACAGTGGGACCTTAGCTT
IRF1	GAACGGACTCTCACTCCAGC	TGGGGGACACCTGAAAGTTG
IFN-β	TGCAGAAGCAAAACTCCACT	GCACACCTGTTGTACTCCTT
ISG15	AAGCAGTTCATCGCCCAGAA	GACCCTTGTCGTTCCTCACC
MIX1	CCACCACCGACAGCTCCCCT	GCAGGTGTGGGCGTGAAGCA

### Overexpression and knockdown

The *Capra hircus* IRF1-AS gene was cloned into the pCDNA3.1 (+) vector (Invitrogen, V790-20). The siRNA against IRF1-AS was synthesized by Ribo Biotechnology. EECs and GFFs grown to 80% confluence in 12-well cell culture plates were transfected with siRNA or pcDNA3.1-IRF1-AS using TurboFect Transfection Reagent (Thermo Fisher Scientific, R0531) according to the manufacturer’s instructions. Then, the cells were cultured in 5% CO_2_ at 37 °C for 24 h. The reaction mixture was discarded, and the cells were then infected with PPRV at the indicated multiplicity of infection (MOI). Nontargeting siRNA (NC siRNA) and pCDNA3.1 were used as a negative control in gene overexpression and RNA interference, respectively.

### Western blot analysis

The harvested cells were treated with RIPA lysis buffer containing phenylmethyl sulfonylfluoride (PMSF) for generating cell lysates. Protein samples were produced by adding 5× SDS-PAGE sample buffer to lysed cells. The samples were boiled for 10 min, separated by 12% sodium dodecyl sulfate-polyacrylamide gel electrophoresis (SDS-PAGE), and then transferred onto 0.22-µm polyvinylidene difluoride membranes (Millipore, Billerica, MA, USA). The membranes were blocked with 5–10% non-fat milk in TBS-Tween 20 for 2 h and then probed overnight at 4 °C with primary antibodies. After washing, the membranes were reacted with HRP-conjugated secondary antibodies. at room temperature for 1 h. At last, the bound antibodies were detected with enhanced chemiluminescence (ECL) immunoblotting detection reagents (Millipore, Billerica, MA, USA). Images were obtained with a CanoScan LiDE 100 scanner (Canon).

### Strand-specific library construction and quality control of RNA readings

The EECs cultures were divided into two groups. The first group was infected with PPRV Nigeria 75/1 at an MOI of 3 (*n* = 3). The second group was kept without infection as a control (*n* = 3). The cells were collected at 48 h post-infection (hpi). Subsequently, total RNA was extracted from 6 samples (three PPRV-infected and three mock-infected samples) using TRIzol reagent (Invitrogen, Carlsbad, CA, USA) according to the manufacturer’s protocol. A NanoPhotometer spectrophotometer (NanoDrop products IMPLEN, CA, USA) was used to measure the quantity and purity of the total RNA, and RNA integrity was tested by the Bioanalyzer 2100 system (Agilent Technologies, CA, USA). Then, mRNAs and lncRNAs were enriched by removing ribosomal RNAs (rRNAs) from qualified total extracted RNA with the Ribo-Zero Magnetic kit (EpiCentre). Enriched mRNAs and lncRNAs were fragmented into short fragments. From these short RNA fragments, first-strand cDNA was synthesized with hexamer random primers, and second-strand cDNA was generated by substituting dTTP with dUTP. The cDNA fragments were then purified and ligated to adapters, and the second-strand cDNA was digested using uracil-*N*-glycosylase (UNG). After agarose gel electrophoresis, suitable fragments were selected as templates for PCR amplification. The final cDNA library quality was assessed on a Qubit 2.0 Fluorometer (Life Technologies, USA) and the Agilent Bioanalyzer 2100 system (Agilent Technologies, USA). Finally, 6 high-quality libraries were obtained and sequenced on an Illumina HiSeq 2500 (Illumina, USA).

### Analysis of sequencing data

Raw reads from each library were produced from RNA sequencing and then filtered by removing adapter reads, low-quality reads and reads containing over 10% Ns. At last, clean reads were obtained. Next, the Q20, Q30, and GC content was monitored to evaluate the clean reads. Subsequently, all the clean reads were aligned were aligned and mapped to the *Capra hircus* reference genome (genome assembly: Capra_hircus GCF_001704415.1_ARS1) using Hisat2 (version 2.0.4) under a spliced mapping algorithm with default parameters [42]. Cufflinks (V2.2.1) [43] was used to reconstruct transcripts and generate the final comprehensive set of transcripts with the mapped reads. To detect the novel transcripts, the assembled transcripts were aligned with reference annotation by utilizing Cuffcompare [43]. The novel transcripts met the following parameters: the length of the transcript was longer than 200 bp, and the exon number was more than 2. To identify lncRNA transcripts, the following transcripts were removed: Transcripts which were shorter than 200 nucleotides and having less than two exons, transcripts which were only present in one sample, and transcripts which were encoding a protein family or were a known mRNA transcript. The Coding-Non-Coding Index (CNCI), Coding Potential Calculator (CPC), and Coding-Potential Assessment Tool (CPAT) were used to evaluate the coding potential of the transcripts. For gene expression analysis, matched reads were calculated and then normalized to RPKM values using RSEM. Gene FPKMs were computed by summing the FPKMs of transcripts in each gene group. Differential expression analysis of two groups was performed using the DESeq R package (1.8.3). *P* value < 0.05 and |log_2_(fold change)|≥1 were set as the thresholds for significantly differential expression by default.

### Target gene prediction

In order to reveal the interactions between lncRNAs and mRNAs, RNAplex software was used to predict the complementary correlation of antisense lncRNAs and mRNAs. One of functions of lncRNAs is the cis-regulation of neighbouring genes on the same allele. The cis-acting target gene predicted that the function of lncRNA was related to the protein-coding genes adjacent to the coordinate. We searched coding genes 100 kb upstream and downstream of lncRNA and then analysed their function next.

### GO and KEGG pathway analyses

Gene ontology (GO) and Kyoto Encyclopedia of Genes and Genomes (KEGG) analyses were performed to identify biological processes and pathways associated with the cis and antisense target genes of the DE lncRNAs and DE mRNAs. A false discovery rate (FDR) was used to correct the *P* values. A corrected *P* value (Q value) < 0.05 was considered significant.

### Immunoprecipitation assay

EECs were transfected for 48 h and incubated on ice with immunoprecipitation lysis buffer (Beyotime, P0013). For each sample, 500 µL of lysate was incubated with 2 µg of antibody and 800 µL of protein A/G plus agarose (Santa Cruz Biotechnology, sc-2003) overnight. The agarose beads were washed 4 times with 1 mL of lysis buffer containing 1% NP-40 (Beyotime, ST366). The precipitates were detected by SDS-PAGE and immunoblotting.

### Confocal immunofluorescence microscopy

Following the indicated treatments, cells were washed 4 times with PBS and fixed in 4% paraformaldehyde. The cells were washed again 4 times with PBS and treated with 0.1% Triton X-100 (Sangon Biotech, A110694) for 15 min. Then, the cells were incubated with 1% bovine serum albumin (BSA; Sigma-Aldrich, A7906) and the appropriate primary antibodies for 1 h at 37 °C before being washed and incubated simultaneously with FITC- or PE-conjugated secondary antibodies. Finally, the cells were treated with a Hoechst 33,342 (Sigma-Aldrich, B2261) solution for 5 min and analysed under a confocal microscope (CLSM; LeicaSP8, Germany).

### Statistical analysis

All values are expressed as the arithmetic mean of triplicates ± standard error of the mean (SEM). Significance was determined by one-way ANOVA with a Dunnett post-test or by paired Student’s *t* test. Values of *P* < 0.05 were considered to indicate statistical significance.

## Results

### PPRV infection activates the innate immune response in EECs

**Figure 1:**
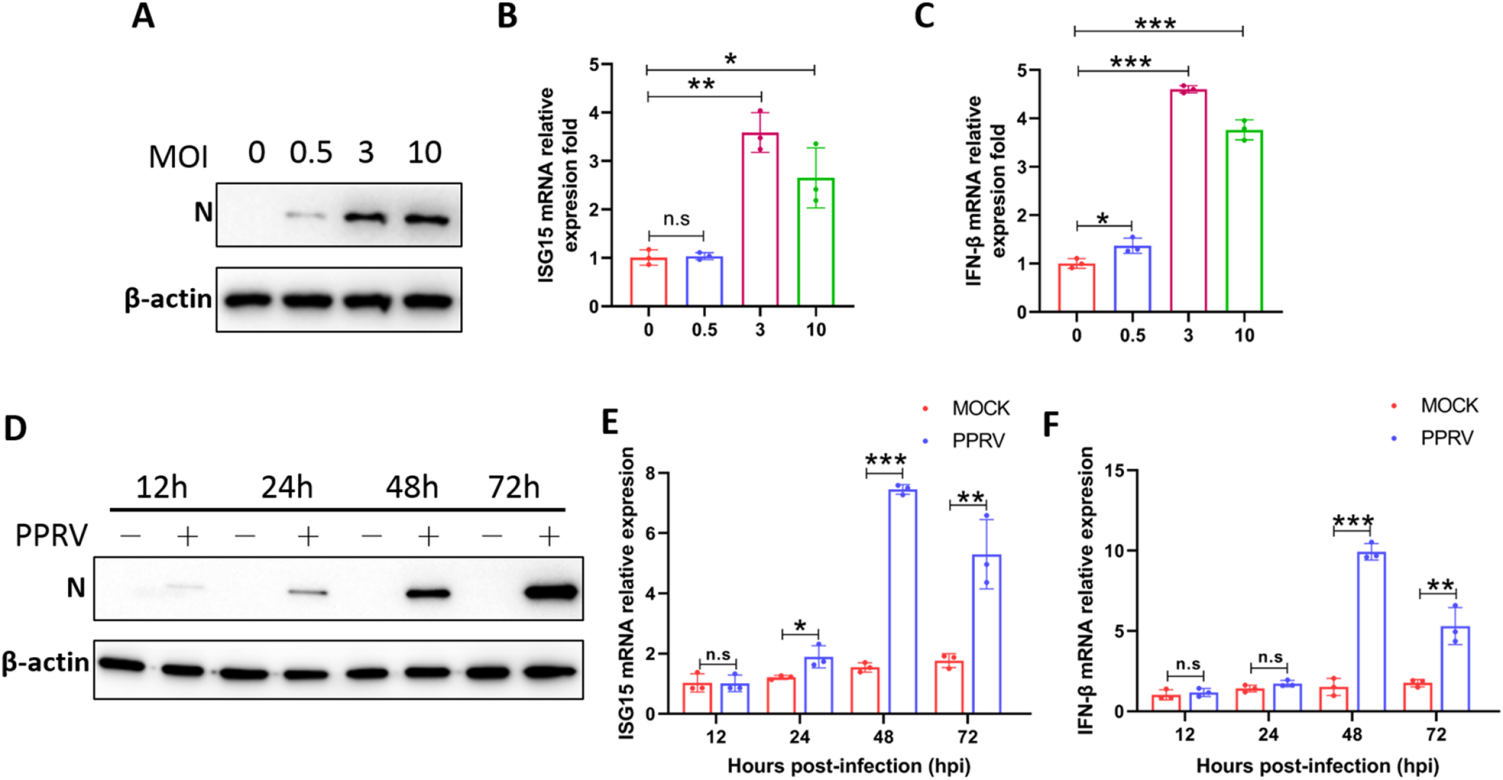
**PPRV infection activates the innate immune response in EECs.** **A–F** EECs were infected with PPRV at different MOIs for 48 h (**A–C**), or at an MOI of 3 for the indicated times (**D–F**), and the protein levels PPRV N expression (**A**, **D**), the mRNA levels of ISG15 (**B**, **E**) as well as Mix1 (**C**, **F**) were measured by Western blot and quantitative RT-PCR assay, respectively. β-actin was used as a loading control in Western blot analysis. The data represent the mean ± SD of three independent experiments. *P* values were calculated using Student’s *t* test. An asterisk indicates a comparison with the indicated control. *P < 0.05; **P < 0.01; ***P < 0.001.

The innate immune system is the first line of defence against microbial invasion. Type I IFN production is significantly involved in host antiviral processes. To study the innate immune response in EECs during PPRV infection, EECs were mock infected or infected with PPRV, and the expression levels of IFN-β and ISG15 were measured. The transcription levels of IFN-β and ISG15 were upregulated in a viral dose- and infection time-dependent manner. At 48 h after infection at an MOI of 3, the IFN-β transcription levels peaked (Figures 1A–C). At an MOI of 3, the expression levels of IFN-β and ISG15 increased rapidly within 24–72 h after PPRV infection, and reached a relative peak at 48 h (Figures 1D–F). These results indicate that the host immune system responds to PPRV infection. Based on these results, to study the role of lncRNAs in defending against PPRV infection by regulating the innate immune response, we harvested PPRV-infected and mock-infected EECs at 48 hpi (MOI = 3) in triplicate for library construction and lncRNA sequencing.

### Overview of sequencing data

**Figure 2:**
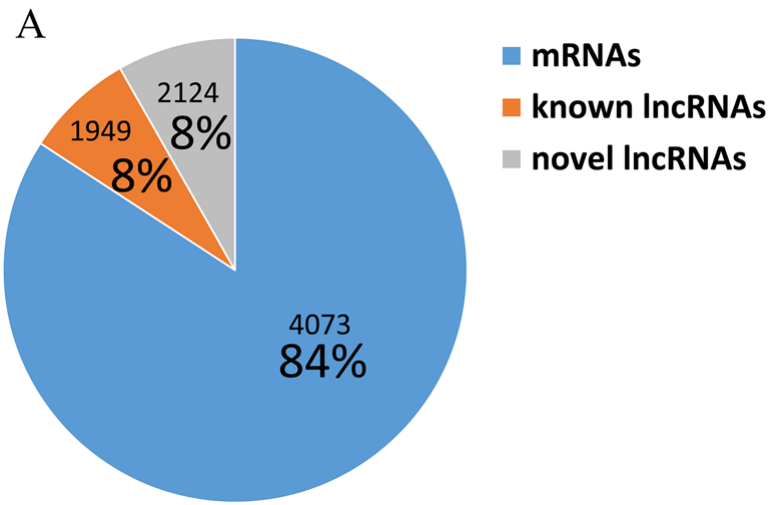
**Venn diagram of classification of transcripts.** **A** The classification and proportion of two main types of assembled transcripts.

Subsequently, 6 libraries were obtained. These libraries were subjected to high-throughput sequencing on the Illumina HiSeq platform. A total of 689.64 M raw reads were acquired from 6 libraries. After removing low-quality sequences and adapter sequences, 669.56 M clean reads were identified in the mock-infected and PPRV-infected groups. The percentage of clean reads with a Phred quality value of more than 30 ranged from 94.48 to 95.20% (Table 2). Overall, 96.38–97.29% of the clean reads aligned with *Capra hircus* (GCF_001704415.1_ARS1) (Table 2). After a series of strict screening conditions, a total of 4073 lncRNAs were identified, including 2124 novel lncRNAs. In addition, 21 685 mRNAs were identified (Figure 2A).

**Table 2:** **Data quality of lncRNA and mRNA profiles**

Sample	Raw reads (M)	Clean reads (M)	Q30 of clean reads (%)	Total mapped reads (%)
M1	112.44	109.29	94.92	97.20
M2	114.94	111.67	94.59	97.15
M3	114.94	111.78	94.50	97.25
P48_1	117.44	113.19	94.57	96.38
P48_2	114.94	111.81	94.48	97.28
P48_3	114.94	111.82	95.20	97.29

### Differential expression of lncRNAs and mRNAs in PPRV-infected versus mock-infected EECs

**Figure 3:**
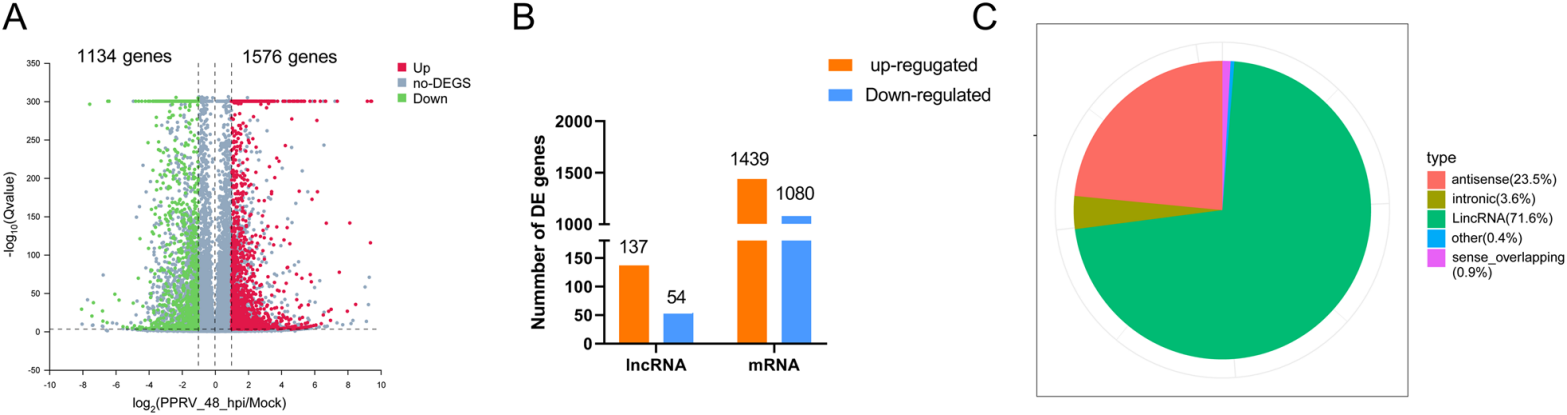
**Differential expression of lncRNAs and mRNAs in PPRV-infected and mock-infected EECs cells**. **A** Volcano plot of differentially expressed mRNAs and lncRNAs. Red dots indicate the upregulated transcripts. Green dots show the downregulated transcripts. Transcripts with less than 1.0-FC and a *P* value more than 0.05 are shown in gray color. **B** Number of differentially expressed mRNAs and lncRNAs. Yellow and blue represent the number of upregulated and downregulated genes, respectively. **C** The classification and proportion of types of identified lncRNAs.

To analyse the differential expression of mRNAs and lncRNAs between the control and PPRV-infected groups, a *P* value < 0.01 and a |log_2_ (fold change)|>1 were used as the cut-off values. Given these criteria, a total of 2710 genes, including lncRNA genes and coding protein genes, were DE in PPRV-infected EECs compared with mock-infected cells (Figure 3A). Of these, 137 lncRNAs were upregulated, and 54 lncRNAs were downregulated (Figure 3B). In addition, 2519 mRNAs showed significantly different expression between the mock- and PPRV-infected cells, including 1439 upregulated and 1080 downregulated mRNAs (Figure 3B). Among these DE lncRNAs, 71.6% were classified as long intergenic noncoding RNAs (lincRNAs), and 23.5% were classified as antisense lncRNAs (Figure 3C). These results revealed that lncRNAs were DE due to viral infection.

### Gene ontology analysis of DE mRNAs

**Figure 4:**
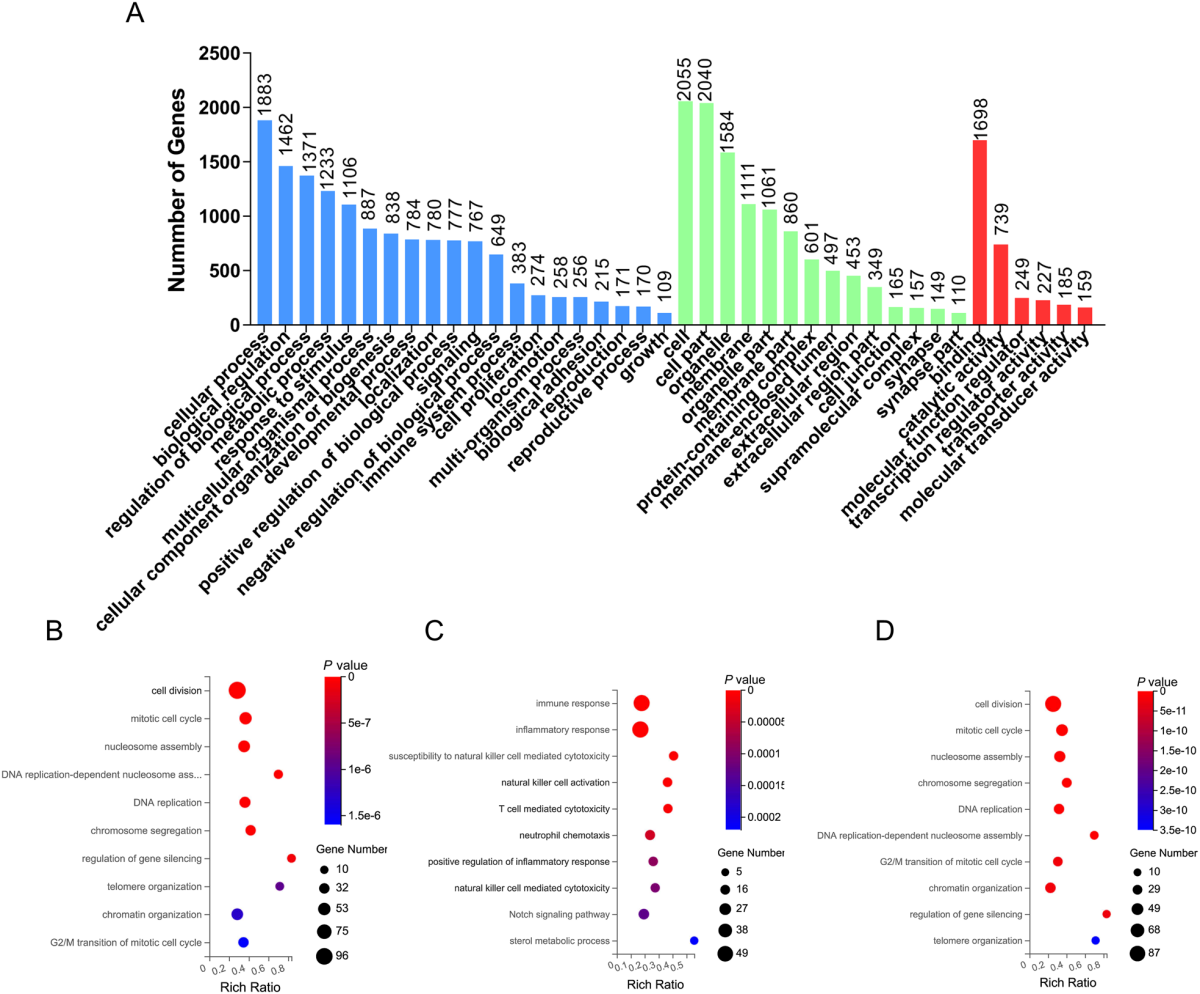
**Gene ontology analysis of differently expressed mRNAs**. **A** GO terms’ classification of the DE mRNAs. **B** Top 10 enriched GO terms in BP, CC, and MF identified by GO analysis. **C** Top 10 enriched GO terms of upregulated mRNAs. **D** Top 10 enriched GO terms of downregulated mRNAs. BP refers to biological process; CC means cellular component, and MF indicates molecular function.

To better understand the potential roles of host factors involved in PPRV infection, the DE mRNAs were subjected to GO annotation analysis. The 2519 DE mRNAs were classified into biological processes, cellular components, and molecular functions (Figure 4A). To further study the potential functions of these DE mRNAs, GO enrichment analysis was performed. The top 10 enriched biological process (BP) GO terms are shown in Figure 4B. The DE mRNAs were enriched in response to cell division, the mitotic cycle and nucleosome assembly (Figure 4B). Furthermore, we analysed the GO enrichment of the upregulated mRNAs (Figure 4C) and downregulated mRNAs (Figure 4D). The upregulated DE mRNAs were generally enriched in response to immune response, inflammatory response and positive regulation of inflammatory response. In contrast, the downregulated DE mRNAs were largely enriched in response to cell division, the mitotic cycle and nucleosome assembly, which was similar to the GO enrichment results for all the DE mRNAs. This finding indicates that the innate immune system might be activated by PPRV in EECs and that immune-related proteins were upregulated to build a line of defence to counteract PPRV infection.

### Bioinformatics analysis of the DE lncRNAs

**Figure 5:**
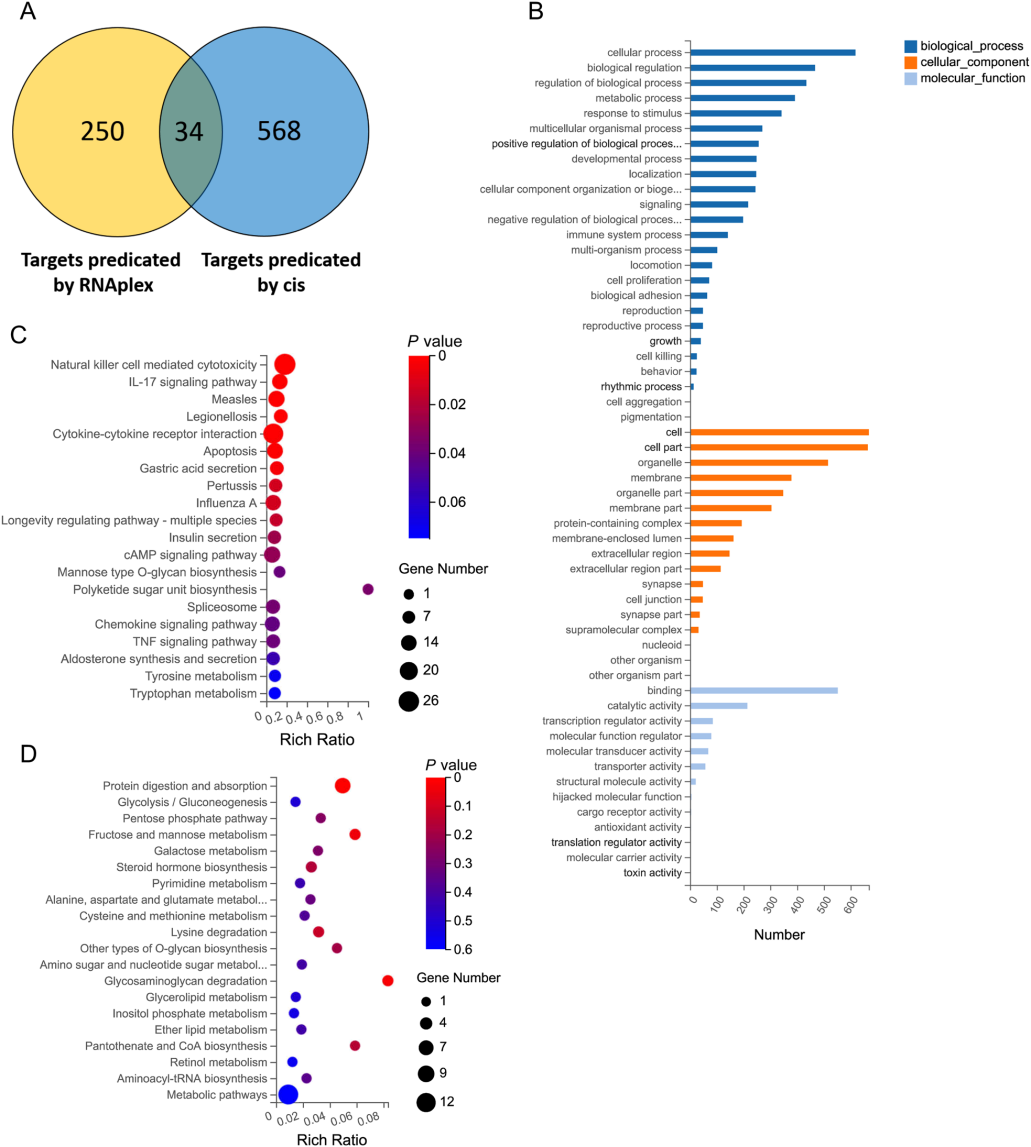
**Bioinformatics analysis of the differentially expressed lncRNAs**. **A** Venn diagram shows the number of overlap genes in DE lncRNAs target genes predicated by cis and RNAplex. **B** GO analysis of the target genes of DE lncRNAs. **C**, **D** KEGG pathway enrichment analysis of target genes. **C** KEGG analysis for upregulated lncRNAs targets. **D** KEGG analysis for downregulated lncRNAs’ targets. Rich Factor refers to the ratio of target gene numbers annotated in this pathway to all identified protein numbers annotated in this pathway. A larger rich factor with a lesser *P* value indicates a greater intensiveness.

lncRNAs play a role in various biological processes by affecting the expression of their neighbouring genes [44]. Therefore, to understand the molecular function and biological processes of lncRNAs during PPRV infection, we searched for protein coding genes within 100 kb of each DE lncRNA as their cis-target genes. As a result, 600 target genes for 162 DE lncRNAs were predicted (Additional file 1). In addition, to study the interactions between antisense lncRNAs and mRNAs, RNAplex software was used to predict the complementary correlation of antisense lncRNAs and mRNAs. It was found that 79 antisense lncRNAs had a complementary relationship with 284 mRNAs, which were considered one part of DE lncRNA targets (Additional file 2). Eventually, a total of 852 target genes for 191 DE lncRNAs in the mock-infected and PPRV-infected groups were predicted (Figure 5A) (Additional file 3). The top 20 (4 DE lncRNAs had no predicated genes) upregulated DE lncRNAs and top 20 (4 DE lncRNAs had no predicated genes) downregulated DE lncRNAs were extracted and are listed (including the DE lncRNA target genes) in Tables 3 and 4, respectively. To explore the potential biological function of DE lncRNAs, GO and KEGG analyses of the target genes of the DE lncRNAs were performed. According to the GO annotation, 852 target mRNAs were classified into 55 GO terms, namely, 25 BP, 17 cellular component (CC) and 13 molecular function (MF) terms (Figure 5B). We found that 141 target mRNAs were classified as the BP term immune system (Additional file 4). Based on the fold change values, 20 target mRNAs that were classified as immune system and their associated 25 DE lncRNAs are listed in Table 5, These target genes include CXCL1, CXCL6, IRF1, PF4, AXL, SLAMF6, HOXB3, MASP1 TOX and so on. Furthermore, the KEGG database was employed to analyse the molecular pathways and cellular processes related to the DE lncRNAs. The targets of the upregulated lncRNAs were associated with immune response-related signalling pathways, such as natural killer cell-mediated cytotoxicity, the IL-17 signalling pathway, cytokine–cytokine receptor interactions, the chemokine signalling pathway and the TNF signalling pathway (Figure 5C). However, targets of the downregulated lncRNAs were involved in protein digestion absorption, gluconeogenesis and the pentose phosphate pathway (Figure 5C). These results reveal that host cells might upregulate lncRNAs to enhance the immune response against PPRV infection.

**Table 3:** **A part of downregulated lncRNAs and their target mRNAs**

Gene ID	Fold change	Targets gene
BGIG9925_32398	− 4.99462	COL21A1
106502872	− 4.6076	TSPYL5, LOC102168924,
BGIG9925_31640	− 4.36659	ARNTL
BGIG9925_31429	− 3.45736	SDCBP, NSMAF, TOX, LOC102181776, TGM6, PNPLA3, LOC102170144
BGIG9925_30294	− 3.44882	DHX15, GPR173, TRIM25, IKZF4, DHRS13, IGSF9, MASP1, PRKN, COLGALT2, DIABLO, SEMA6A
106501953	− 3.40359	CCN1, ZNHIT6
BGIG9925_29676	− 3.36659	IL22RA1, PNRC2, SRSF10, MYOM3, TAF9B, LOC102189337, ODF3B, TMEM267, PROX2, ZNF502, EDARADD
BGIG9925_30838	− 3.18018	TNPO2, NAA15, GFRA1, USP15, INHBA
108638305	− 3.07709	MLX, PSMC3IP, CAVIN1, NAGLU, HSD17B1, TUBG1, ATP6V0A1, COASY, RETREG3
BGIG9925_29952	− 3.01137	ALX3, CSF1, STRIP1, AHCYL1
106502031	− 2.99233	CAV1, CAV2
BGIG9925_32563	− 2.88698	PDGFA, GPER1, PRKAR1B, GPR14
BGIG9925_32056	− 2.80119	ADAMTS6, CWC27, SLAMF6, LOC102185066, EXOC4, LOC108634317, TSC22D3, FAM216B, GABPB2
106503672	− 2.79574	MFHAS1, ERI1,
106502189	− 2.71452	LGI2, CCDC149, CCDC149

**Table 4:** **A part of upregulated lncRNAs and their target mRNAs**

Gene ID	Fold change	Targets gene
106502218	5.950819	PPBP, CXCL6, PF4, CXCL1,
108635918	5.55227	LOC102174202, LOC102176015
108636242	4.414767	ZFYVE28, HAUS3, POLN, MXD4, CFAP99
108637009	4.180301	FBXO42, ARHGEF19, EPHA2, CPLANE2
108636281	4.047035	PPBP, LOC102181582, LOC102181854, LOC102182115, CXCL8, LOC108636232
BGIG9925_30908	3.900193	ATN1, LOC102191423, LOC102178686
108635850	3.736695	ZNRF2, GGCT, NOD1
108637526	3.55227	ZC2HC1A, IL7, GRXCR2
108636925	3.307851	LOC102175964, LOC102187597, LOC102188051, LOC102188506, ERG28, TTLL5
BGIG9925_31100	3.226313	DCT, TGDS, GPR180, PNPLA3
106502447	3.151732	LOC102169960, LOC102170234, NIPSNAP3A, ABCA1
BGIG9925_29876	2.900193	LMO4, ERBB3
108638258	2.900193	VTN, IFT20, TNFAIP1, TMEM199, NLK, TMEM97, POLDIP2, SEBOX, SARM1, SLC46A1, MIIP, TNFAIP1
102185883	2.747703	IGSF8KCNJ9, COPA, PEX19, DCAF8, PEA15, CASQ1, LOC102186069, LOC102186539, KCNJ10’
BGIG9925_29643	2.727789	TIPARP, SSR3, CHD2
108637780	2.677801	LRRC47, CCDC27, C16H1orf174, DFFB CEP104, SMIM1, TP73, CEP104

**Table 5:** **A part of lncRNA-mRNA pairs related to immune system**

lncRNA ID	lncRNA Fold change	mRNA ID	mRNA name	Mrna Fold change
106502218	5.9508195	102182115	CXCL1	8.135882
108636281	4.0470348			
108636281	4.0470348	102178438	CXCL8	7.382056
106502218	5.9508195	102182683	LOC102182683	6.680369
102177673	1.5250821	102175816	CFB	5.40409
106502218	5.9508195	102181582	CXCL6	5.21843
108636281	4.0470348			
BGIG9925_33000	1.3361411	108634235	LOC108634235	4.414767
106502306	2.3152309	102171507	FCER2	3.999729
106502218	5.9508195	102,181,854	PF4	3.677801
108636281	4.0470348			
108636069	− 2.323372	102173256	PIK3CG	2.900193
108636385	1.6474273	102188583	IRF1	2.20066
106503007	1.220594	102169209	LOC102169209	− 1.2062
102186229	− 1.366593	102191074	AXL	− 1.36096
BGIG9925_29643	2.7277891	102182170	TIPARP	− 1.38627
BGIG9925_32056	− 2.801192	102181738	SLAMF6	− 1.49212
106501937	1.0103763	102181629	BTLA	-1.49212
BGIG9925_30879	1.3823451	102176966	ACTN1	− 1.70949
BGIG9925_31429	− 3.457359	102188688	TOX	− 1.76572
108636333	1.501644	102171378	MASP1	− 2.44358
BGIG9925_30294	− 3.44882			
102169699	− 1.09027	102169623	HOXB3	− 2.78532
106503278	− 1.074059			
102177673	1.5250821	102179996	MPIG6B	− 3.71452

### Validation of deep sequencing results by qPCR

**Figure 6:**
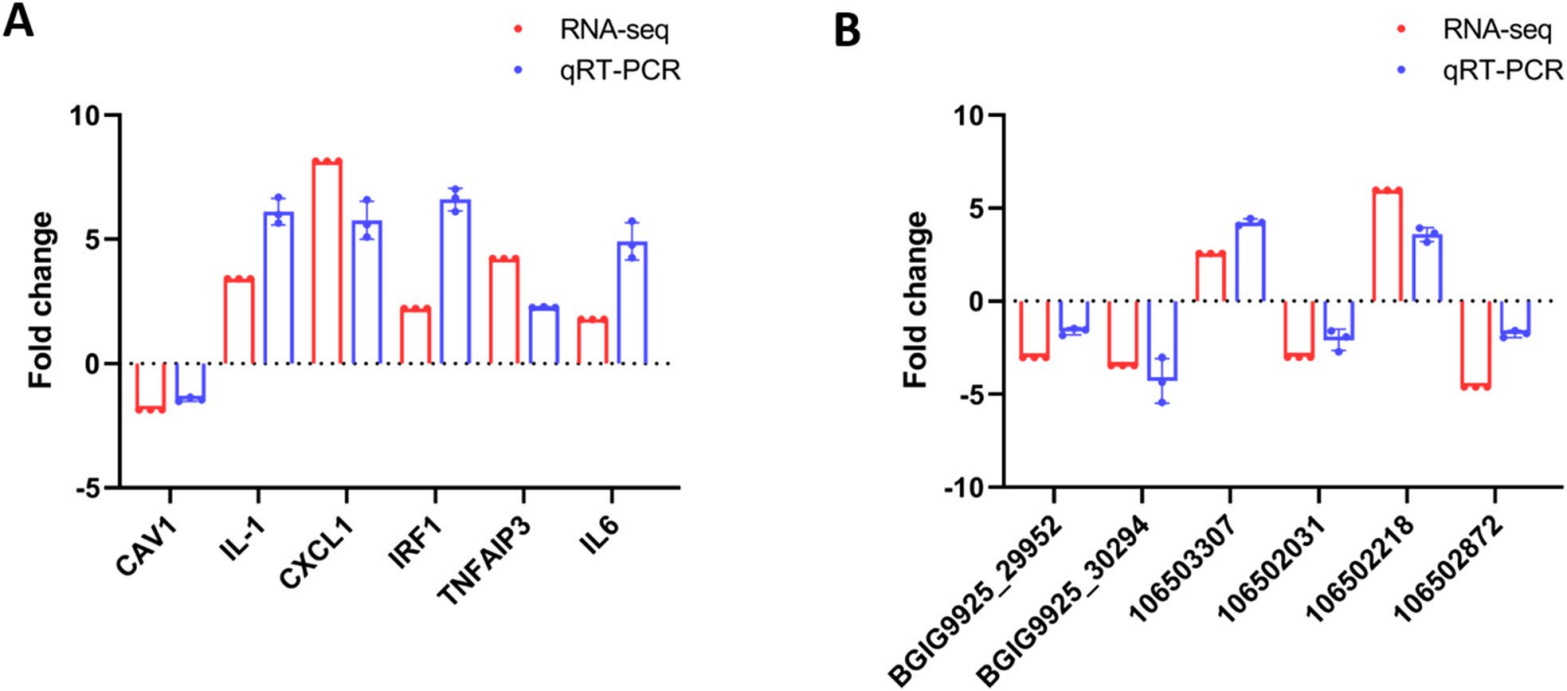
**Validation of deep sequencing results by qPCR**. **A**, **B** The relative expression level of each and mRNA (**A**) and lncRNA (**B**) in PPRV-infected EECs were calculated using the 2^−ΔΔCT^ method and represented as the n-fold change compared to the mock-infected sample. The gene actin was used as the reference gene.

To verify the credibility of the expression profiles of the mRNAs and lncRNAs in PPRV-infected EECs obtained from the RNA sequencing analysis, six DE lncRNAs and six mRNAs were randomly selected for qRT–PCR analysis. As a result, the relative expression levels of the selected lncRNAs and mRNAs showed similar trends to the sequencing data (Figure 6). Therefore, the data obtained from sequencing were considered reliable.

### Identification of PPRV-induced IRF1-AS

**Figure 7:**
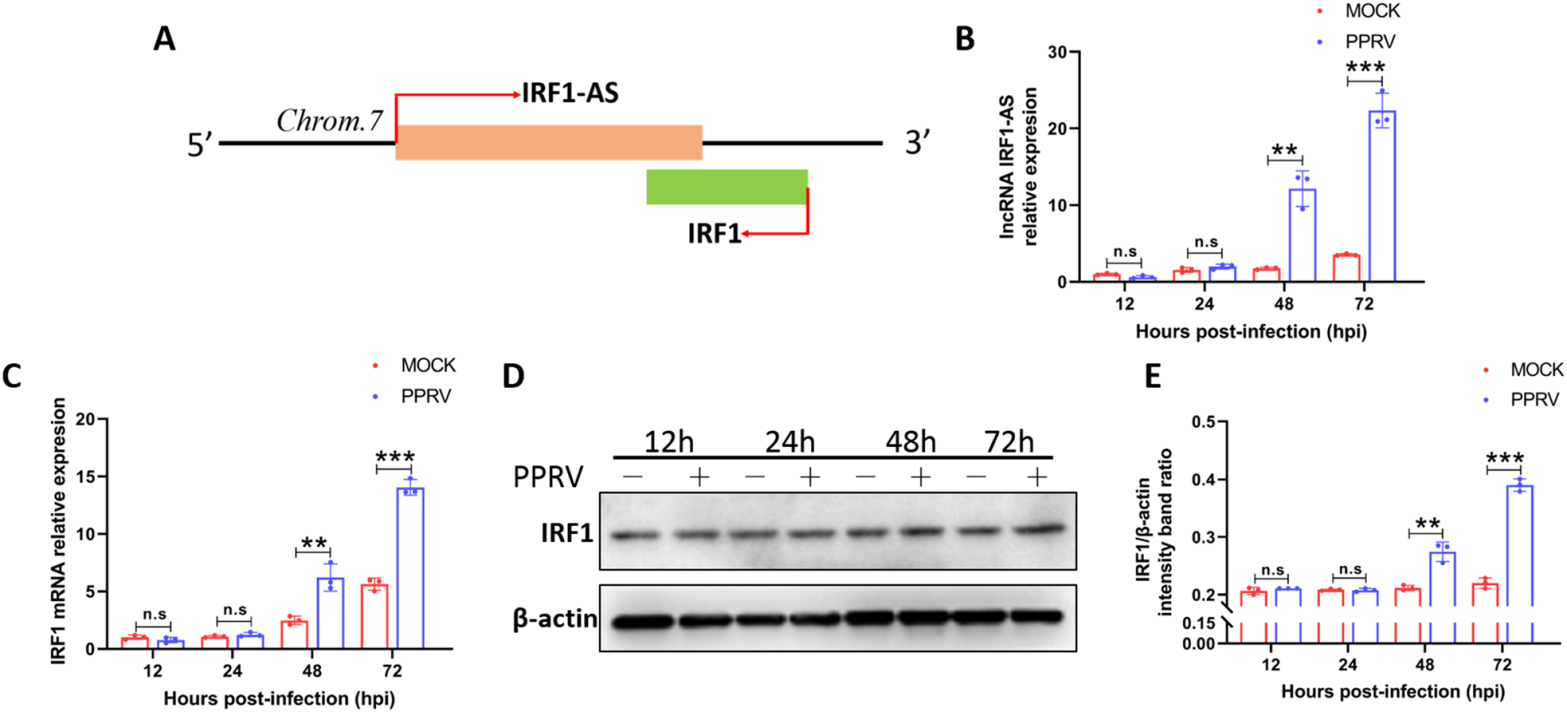
**Identification of PPRV-induced IRF1-AS**. **A** Schematic representation of the IRF1-AS and IRF1 locus in the *Capra hircus* genome. **B**–**D** EECs were infected with PPRV at an MOI of 3 for the indicated times, and the transcription levels of IRF1-AS (**B**), IRF1 (**C**) as well as the protein levels of IRF1 (**D**), were measured by quantitative RT-PCR and Western blot assay, respectively. **E** The relative quantification of IRF1 protein levels compared to β-actin protein levels was determined by densitometry. β-actin was used as a loading control in Western blot analysis. The data represent the mean ± SD of three independent experiments. *P* values were calculated using Student’s *t* test. An asterisk indicates a comparison with the indicated control. n.s., no significant; **P < 0.01; ***P < 0.001.

According to Table 5, we found an RNA annotated as lncRNA 108,636,385, whose target mRNA was IRF1. We noticed that lncRNA 108,636,385 was transcribed from the antisense strand in the opposite direction relative to IRF1 (Figure 7A); therefore, we named it interferon regulatory factor 1 antisense (IRF1-AS) RNA. Subsequently, we investigated the possible relationship between IRF1-AS and IRF1 by examining their correlation in EECs. Our data showed that IRF1 is upregulated at both the mRNA and protein levels, accompanied by IRF1-AS upregulation during PPRV infection (Figures 7B–E), which confirmed a positive correlation between IRF1-AS and IRF1 levels. IRF1 is an extensively characterized ISG and a central regulator of the IFN response [45]. As IRF1-AS is also upregulated during viral infection, we investigated the function and mechanism of IRF1-AS in PPRV replication as well as its correlation with IRF1.

### IRF1-AS downregulation enhances PPRV replication

**Figure 8:**
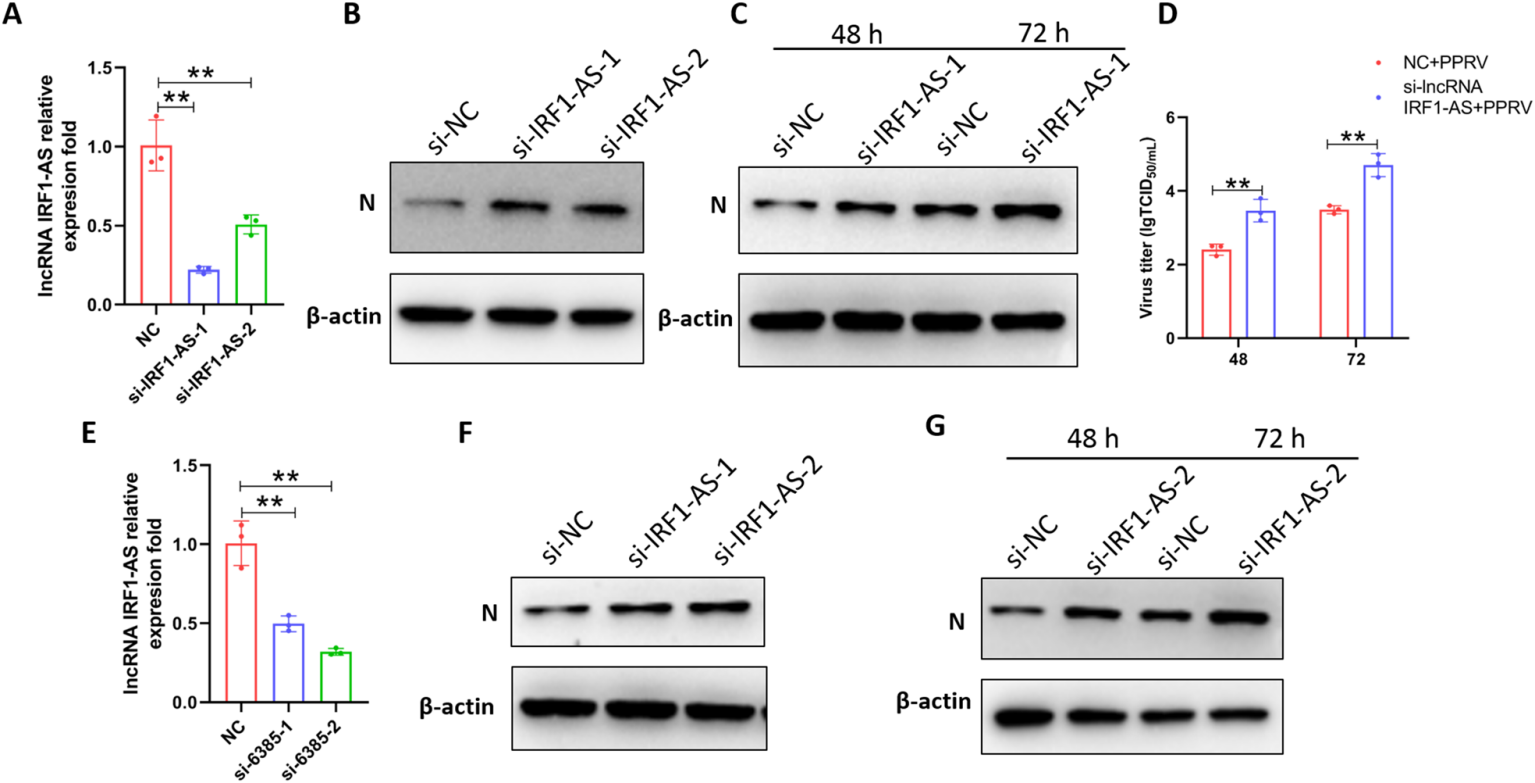
**IRF1-AS downregulation enhances PPRV replication**. **A**, **B** EECs and **E**, **F** GFFs were transfected with si-NC or si-IRF1-AS-1 and si-IRF1-AS-2 siRNA for 24 h and then infected with PPRV at an MOI of 3 for 48 h. The RNA expression levels of IRF1-AS (**A**, **E**) and the protein levels PPRV N expression (**B**, **F**) were measured by quantitative RT-PCR and Western blot assay, respectively. **C** EECs and **G** GFFs were transfected with si-NC or si-IRF1-AS-1 for 24 h and then infected with PPRV at an MOI of 3 for the indicated times. The protein levels PPRV N expression were measured by Western blot assay. **D** EECs were transfected with si-NC or si-IRF1-AS-1 for 24 h and then infected with PPRV at an MOI of 3 for the indicated times. In the indicated time, the virus titres in the supernatants were measured by TCID_50_ assay. β-actin was used as a loading control in Western blot analysis. The data represent the mean ± SD of three independent experiments. *P* values were calculated using Student’s *t* test. An asterisk indicates a comparison with the indicated control. n.s., no significant; **P < 0.01.

To evaluate the influence of IRF1-AS on PPRV proliferation, we performed a small interfering RNA (siRNA) transfection assay to downregulate the expression of IRF1-AS in EECs and GFFs. EECs and GFFs were transfected with siRNA against IRF1-AS or NC siRNA for 24 h and then infected with PPRV. At 48 h post-infection, cell samples were collected to measure IRF1-AS levels using qRT–PCR. After transfection with si-IRF1-AS-1 and si-IRF1-AS-2, endogenous IRF1-AS expression at the transcriptional level was significantly reduced, while IRF1-AS expression was most obvious for si-IRF1-AS-1 in EECs (Figure 8A) and for si-IRF1-AS-2 in GFFs (Figure 8E). In contrast, knockdown of IRF1-AS considerably enhanced PPRV N protein expression levels, while N protein expression was most obvious for si-IRF1-AS-1-transfected EECs (Figure 8B) and for si-IRF1-AS-2-transfected GFFs (Figure 8F). Moreover, EECs were transfected with si-IRF1-AS-1 or NC siRNAs and then infected with PPRV at 3 MOI at 24 h post-transfection. At 48 and 72 hpi, the cells were harvested for TCID_50_ and Western blot assays. We found that compared with the cells transfected with NC siRNAs, those with knockdown of IRF1-AS exhibited considerably enhanced PPRV protein expression levels and viral yields at different time points (Figures 8C and D). In addition, we transfected GFFs with si-IRF1-AS-2 and harvested them at 48 and 72 hpi to detect N protein by Western blotting. The results showed similar trends to those for EECs. These results clearly show that knockdown of endogenous IRF1-AS significantly promotes PPRV replication.

### IRF1-AS overexpression inhibits PPRV infection

**Figure 9:**
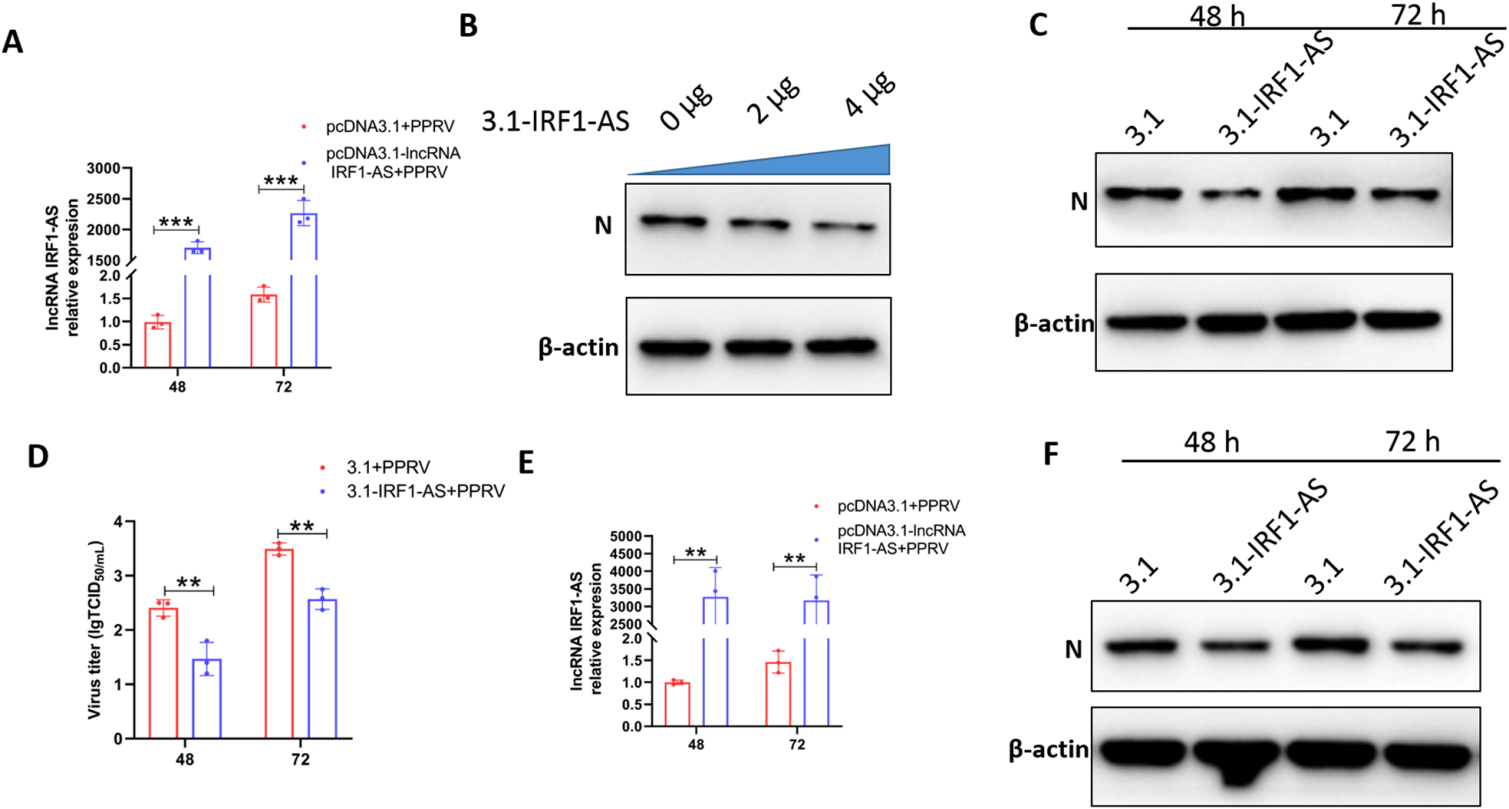
**IRF1-AS overexpression inhibits PPRV replication**. **A**, **C** EECs and **E**, **F** GFFs were transfected with pCDNA3.1 plasmids or pCDNA3.1-IRF1-AS plasmids for 24 h and then infected with PPRV at an MOI of 3 for the indicated times. The RNA expression levels of IRF1-AS (**A**, **E**) and the protein levels PPRV N expression (**C**, **F**) were measured by quantitative RT-PCR and Western blot assay, respectively. **B** EECs were transfected with increasing amounts of pCDNA3.1-IRF1-AS plasmids (0, 2, 4 µg) for 24 h, and the cells were infected by PPRV (MOI = 3) for 48 h. The protein levels of PPRV N were measured by Western blot assay. **D** EECs were transfected with pCDNA3.1 plasmids or pCDNA3.1-IRF1-AS plasmids for 24 h and then infected with PPRV at an MOI of 3 for the indicated times. At the indicated time, the virus titres in the supernatants were measured by TCID_50_ assay. β-actin was used as a loading control in Western blot analysis. The data represent the mean ± SD of three independent experiments. *P* values were calculated using Student’s *t* test. An asterisk indicates a comparison with the indicated control. **P < 0.01; ***P < 0.001.

To further evaluate the effect of IRF1-AS on PPRV replication, we evaluated the PPRV replicative status in IRF1-AS-overexpressing EECs and GFFs. First, EECs and GFFs were transfected with pcDNA3.1-IRF1-AS or pcDNA3.1(+), followed by PPRV infection. The RNA levels of IRF1-AS were detected at different times by qRT–PCR and showed that IRF1-AS expression was significantly upregulated in pcDNA3.1-IRF1-AS-transfected EECs (Figure 9A) and GFFs (Figure 9E) compared with pcDNA3.1(+)-transfected cells. Subsequently, the transfected cells were harvested for PPRV detection. As shown in Figure 9B, IRF1-AS overexpression decreased N protein expression in a dose-dependent manner at 48 hpi in EECs. In addition, IRF1-AS overexpression also decreased N protein expression and titres at 48 and 72 hpi (Figures 9C and D). Furthermore, in GFFs (Figure 9F), IRF1-AS overexpression decreased N protein expression at 48 and 72 hpi. Collectively, these results suggest that IRF1-AS negatively regulates PPRV replication in EECs and GFFs.

### IRF1-AS positively regulates type I IFN production and ISGS expression during viral infection

**Figure 10:**
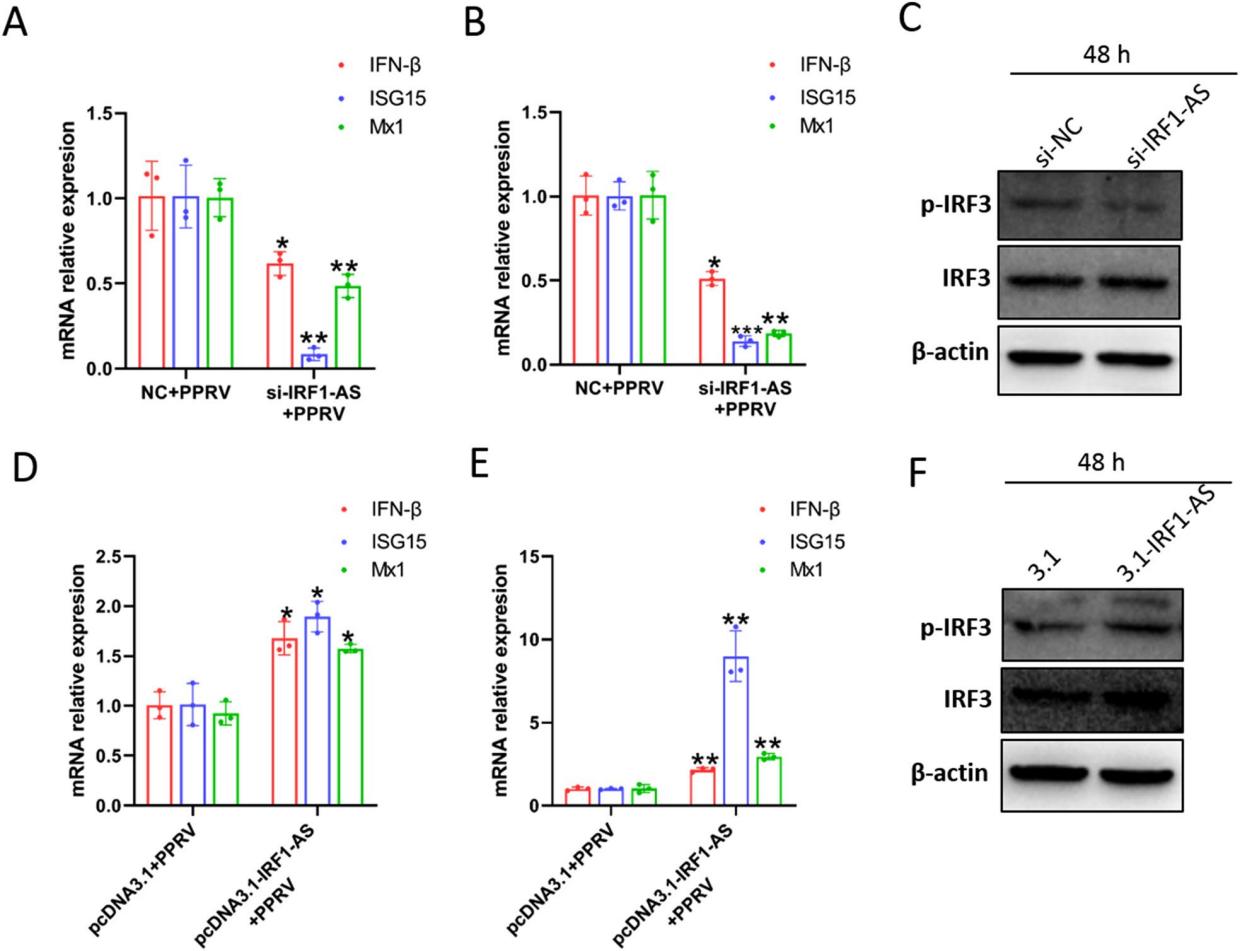
**IRF1-AS positively regulates type I IFN production and ISGS expression during viral infection**. **A** EECs and **B** GFFs were transfected with si-NC or si-IRF1-AS-1 siRNAs for 24 h and then infected with PPRV at an MOI of 3 for 48 h. The RNA expression levels of IFN-β, ISG15 as well as Mix1 were measured by quantitative RT-PCR. **D** EECs and **E** GFFs were transfected with pCDNA3.1 plasmids or pCDNA3.1-IRF1-AS plasmids for 24 h and then infected with PPRV at an MOI of 3 for 48 h. The RNA expression levels of IFN-β, ISG15 as well as Mix1 were measured by quantitative RT-PCR. **C** EECs were transfected with pCDNA3.1 plasmids or pCDNA3.1-IRF1-AS plasmids for 24 h and then infected with PPRV at an MOI of 3 for 48 h. The protein level of IRF3 and phosphorylation of IRF3 were measured by Western blot assay. **F** EECs were transfected with si-NC or si-IRF1-AS siRNA for 24 h and then infected with PPRV at an MOI of 3 for 48 h. The protein level of IRF3 and phosphorylation of IRF3 were measured by Western blot assay. The data represent the mean ± SD of three independent experiments. *P* values were calculated using Student’s *t* test. An asterisk indicates a comparison with the indicated control. *P < 0.05; **P < 0.01.

According to the results above, IRF1-AS exhibited an antiviral function against PPRV. lncRNAs have been found to play important roles during viral infection and the antiviral immune response [31, 46]. Therefore, we speculated that IRF1-AS positively regulates the host innate immune response to suppress viral replication. To assess whether IRF1-AS has a regulatory function in virus-induced IFN-β production, endogenous IRF1-AS expression was downregulated by transfection with a specific siRNA targeting IRF1-AS or upregulated by transfection with pcDNA3.1-IRF1-AS. IFN-β production and ISG expression levels were measured in IRF1-AS-knockdown cells by qRT–PCR. The silencing of IRF1-AS decreased the production of ISG15 and MX1 in PPRV-infected EECs (Figure 10A) and GFFs (Figure 10B). In contrast, the overexpression of IRF1-AS upregulated the expression of ISG15 and MX1 in PPRV-infected EECs (Figure 10D) and GFFs (Figure 10E). The induction of IFN production by viral infection relies on the activation of IRF3. Therefore, we also measured the effect of IRF1-AS on the phosphorylation of IRF3 by Western blotting. The data revealed that the phosphorylation of IRF3 was decreased in IRF1-AS-knockdown cells (Figure 10C) and enhanced in IRF1-AS overexpressed cells (Figure 10F) remarkably during PPRV infection.

### IRF1 enhances the innate immune response and inhibits PPRV replication

**Figure 11:**
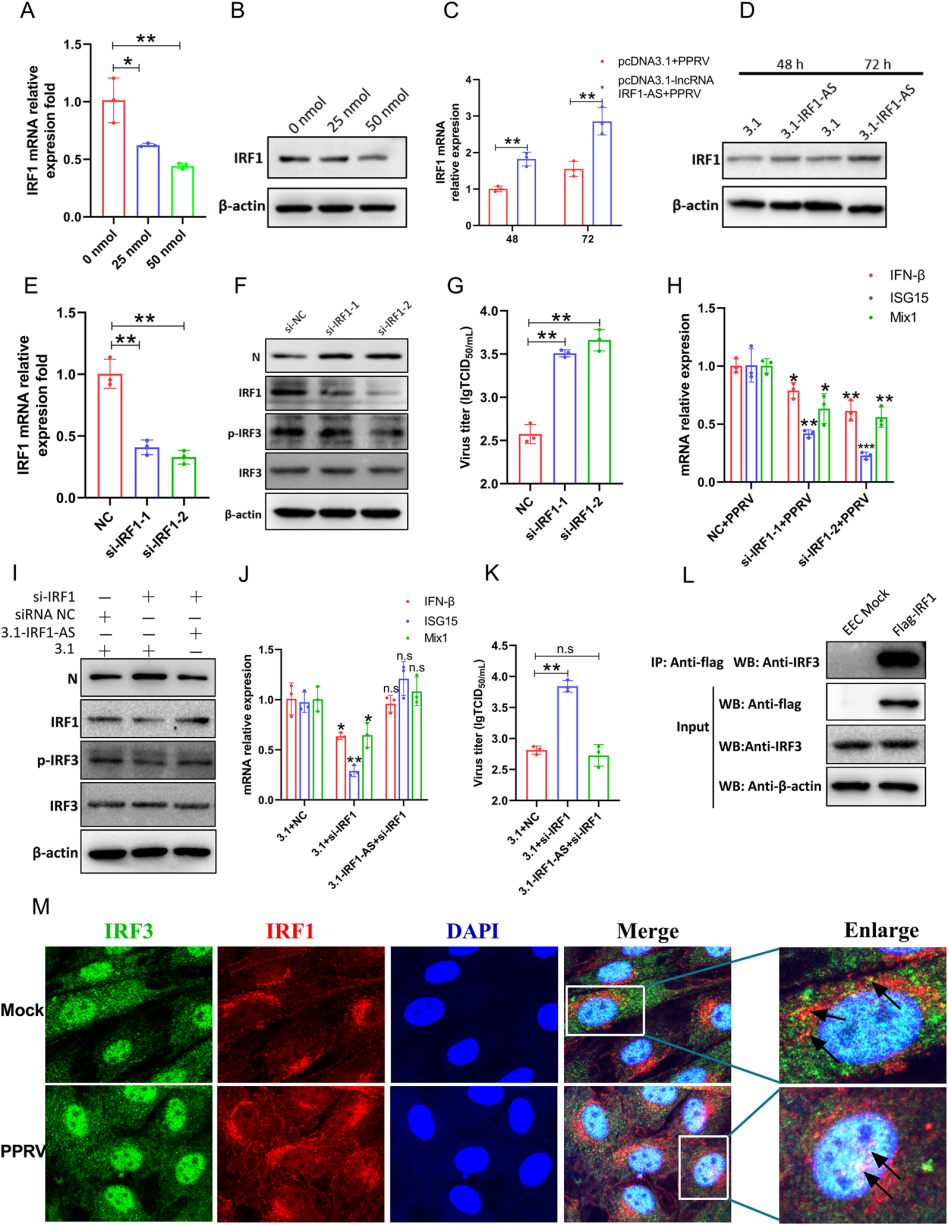
**IRF1 enhances the innate immune response and inhibits PPRV replication**. EECs were transfected with increasing amounts of si-IRF1-AS-1 (0, 25, 50 nmol) (**A**, **B**) or pCDNA3.1-IRF1-AS plasmids (**C**, **D**) for 24 h, and then the cells were infected by PPRV. The mRNA expression levels (**A**, **C**) and the protein level (**B**, **D**) of IRF1 were measured by qRT-PCR and Western blot assay, respectively. EECs were transfected with si-NC or si-IRF1-1 and si-IRF1-2 for 24 h and then infected with PPRV (**E–H**). The RNA expression levels of IRF1 (**E**), IFN-β, ISG15 as well as Mix1 (**H**) were measured by qRT-PCR, the protein level of IRF1, IRF3 and phosphorylation of IRF3 were measured by Western blot assay and the virus titres were measured by TCID_50_ assay (**F**). **I**–**K** EECs were co-transfected with siRNA NC or si-IRF1 and pcDNA3.1 empty vector as control group and 3.1-IRF1-AS and si-IRF1 as the rescue group for 24 h, and then infected with PPRV. **I** The protein level of IRF1, IRF3 and phosphorylation of IRF3 were measured by Western blot assay. **J** The RNA expression levels of IRF1, IFN-β, ISG15 as well as Mix1 were measured by qRT-PCR. **K** The virus titres in the supernatants were measured by TCID_50_ assay. **L** EECs were transfected with pcDNA3.1 empty vector or pcDNA3.1-flag-IRF1. After 24 h, transfected cells were infected by PPRV and then sample were prepared for co-IP experiment. **M** The subcellular localization of endogenous IRF1 and IRF3 was analysed by fluorescence microscopy in EECs infected with PPRV or not. The arrows highlight the nuclear colocalization of IRF3 and IRF1 by immunofluorescence staining. *P* values were calculated using Student’s *t* test. An asterisk indicates a comparison with the indicated control. *P < 0.01; **P < 0.001.

To explore the mechanism by which IRF1-AS inhibits viral replication, we investigated IRF1, a potential target involved in regulating ISG expression. To further investigate the interplay between IRF1-AS and IRF1, we knocked down and overexpressed IRF1-AS in EECs. Both the mRNA and protein levels of IRF1 were decreased in the IRF1-AS-knockdown cells (Figures 11A and B) and increased in the IRF1-AS-overexpression cells (Figures 11C and D). IRF1 is an extensively characterized ISG and a central regulator of the IFN response [45]. We hypothesized that IRF1 may play an important role in the innate immune response to viral infection. To assess this hypothesis, we silenced IRF1 in EECs using siRNA and measured the levels of IFN-β, ISG15 and Mix1 by qRT–PCR. IRF1 was knocked down effectively in EECs at both the RNA (Figure 11E) and protein levels (Figure 11F). Our data indicated that the induction of IFN-β, ISG15 and Mix1 was attenuated (Figure 11H) in IRF1-silenced cells. In addition, we measured the effect of IRF1 on the phosphorylation of IRF3 by Western blotting. The data revealed that the phosphorylation of IRF3 was decreased in PPRV-infected EECs when IRF1 was knocked down with specific siRNA (Figure 11F). The enhanced innate immune response by IRF1 prompted us to further explore the role of IRF1 in the cellular antiviral response. The replication of PPRV was promoted in IRF1-silenced EECs. The results showed that knockdown of IRF1 enhanced the levels of PPRV N protein (Figure 11F) and the viral titres (Figure 11G). To further confirm that IRF1-AS affects PPRV replication mainly by regulating IRF1 expression, we co-transfected cells with siRNA NC or si-IRF1 and pcDNA3.1 empty vector. In the rescue group, si-IRF1 and 3.1-IRF1-AS were co-transfected into cells. After 24 h, cells were infected by PPRV for 48 h. The cell lysates were harvested to evaluate the phosphorylation of IRF3, N protein expression and ISGs production. Our results indicated that silencing IRF1 reduced the phosphorylation of IRF3 and ISGs production, while, increased protein expression of N and viral titres. Furthermore, IRF1-AS overexpression abolished the decrease in the phosphorylation of IRF3 and ISGs production and rescued the enhancement in protein expression of N and viral titres (Figures 11I–K).

IRF1 has been reported to interact with IRF3 to enhance its activation during viral infection [47]. Based on the effect of IRF1-AS and IRF1 on the activation of IRF3 described above, we speculated that IRF1 may function by interacting with IRF3 during PPRV infection. Therefore, to test our hypothesis, we examined the ability of these proteins to form a complex in co-immunoprecipitation (co-IP) experiment. Our data showed that IRF3 was present in the immunoprecipitates obtained with an anti-flag antibody (Figure 11L). The immunofluorescence data also demonstrated that IRF1 colocalized with IRF3 in the cytoplasm of mock cells. IRF3 translocated to the nucleus and colocalized with IRF1 in the nucleus after PPRV infection (Figure 11M). Taken together, IRF1-AS might enhance the innate immune response by promoting the IRF1 interaction with IRF3.

## Discussion

PPRV infection often causes foetal mummification and abortion, resulting in great economic losses in goat and sheep production [10, 11, 48]. The innate immune response is the first line of defence against viruses [49–51]. However, how the uterus subjected to PPRV uses the innate immune response against intracellular pathogen invasion is obscure. In particular, the possible role of lncRNAs in this process is unknown. As a powerful research tool, transcriptome analysis has been widely used to reveal the interaction between host and virus. Recently, many studies have suggested that host-encoded lncRNAs play key roles in regulating the immune response against viral infection [28–30]. Our published studies have confirmed that PPRV can successfully replicate in caprine EECs, further confirming the clinical phenomenon of abortions in PPRV-infected goats [19]. For this reason, to explore the role of lncRNAs in PPRV infection and innate immune regulation, we identified DE lncRNAs in EECs in response to PPRV infection by using the RNA-seq platform. We first determined the innate immune response of EECs during PPRV infection and found that PPRV induced the most obvious innate immune response in EECs at 48 hpi with an MOI of 3. Interestingly, our data also revealed that PPRV infection did not upregulate the expression levels of IFN-β and ISG15 at 12 hpi and 24 hpi. Reasonably, we speculated that PPRV might induce immunosuppression in EECs in the early stage of infection. Zhu’s findings might corroborate our results to some extent [37]. Accordingly, we chose 48 hpi as the time point to measure the lncRNA expression profiles. Detailed analysis revealed many differences in the global expression profile of lncRNAs among mock- and PPRV-infected EECs. A total of 191 DE lncRNAs and 2519 DE mRNAs were identified in the PPRV-infected group compared to the mock-infected group.

To some extent, the function of lncRNAs can be inferred from their associated cis-regulated and trans-regulated mRNAs [52], and the changes in lncRNA expression following viral infection are predicted to have profound effects on host responses [29, 32, 34]. To evaluate the potential biological roles of DE lncRNAs expressed in EECs in response to PPRV infection, we predicted the potential target genes for the DE lncRNAs identified in our lncRNA sequencing datasets. Therefore, we not only searched for protein-coding genes within 100 kb of each DE lncRNA as cis-target genes but also used RNAplex software to predict target mRNAs as trans target genes. As a result, 852 target genes for 191 DE lncRNAs in the mock-infected and PPRV-infected groups were predicted. Using the predicted target genes, the annotation analysis revealed that 141 target mRNAs were classified based on the immune system term. In the KEGG pathway analysis, most of the target genes of the upregulated DE lncRNAs were involved in immune response-related signalling pathways, such as natural killer cell-mediated cytotoxicity, the IL-17 signalling pathway, cytokine–cytokine receptor interactions, the chemokine signalling pathway and the TNF signalling pathway. Additionally, we found that the upregulated mRNAs participated in the immune response, inflammatory response and positive regulation of the inflammatory response, which was similar to the results for the DE lncRNAs. This result indicated that PPRV infection could contribute to the initiation of the host antiviral response and the restriction of PPRV replication. All these functional analyses suggest that the innate immune system might be activated by PPRV in EECs and that immune-related proteins were upregulated to build a line of defence to counteract PPRV infection. By regulating target genes, cellular lncRNAs have a profound effect on the regulation of the innate immunity of EECs to PPRV infection.

The innate immune response plays a vital role in the first line of defence against viruses [49–51]. Type I IFNs, primarily IFN-α/β, are produced by host cells as “early” antiviral agents [53, 54]. Recently, lncRNAs have been shown to be involved in antiviral responses by regulating ISG expression. For example, loc107051710 has an antiviral role during infectious bursal disease virus infection due to enhancement of interferon production [55]; lnc-ISG20 inhibits influenza A virus replication by enhancing ISG20 expression [29]; and Chen and colleagues demonstrated that the lncRNA NRAV modulates antiviral responses through suppression of ISG transcription [31]. Although an important role of lncRNAs in enhancing IFN-induced antiviral effects has been identified, whether lncRNAs participate in PPRV-mediated augmentation of IFN-I-mediated antiviral responses remains elusive. Here, we identified many immune-related lncRNAs (Additional file 4) in PPRV-infected EECs, which was consistent with previous reports in spleen and lung tissues of goats infected with PPRV [56]. Furthermore, 25 DE lncRNAs were identified based on the fold change values of their targeted genes and their roles in immune response. Among these target genes, chemokines (CXCL1, CXCL6 and CXCL8) are low-molecular-weight proteins that belong to the cytokine superfamily and induce immune cell trafficking by binding to their corresponding receptors [57]; IRF1 is an extensively characterized ISG and a central regulator of the IFN response [45]; Chemokine PF4 Inhibits EV71 and CA16 Infections at the Entry Stage [58]; AXL, a receptor tyrosine kinase, promotes Zika virus infection in astrocytes by antagonizing type I interferon signalling [59]; MASP1, a multifunctional serine protease of complement and coagulation, plays a central role in the early innate immune response [60] Moreover, we found that EECs could enhance IFN-mediated antiviral responses upon PPRV infection by inducing cellular IRF1-AS to inhibit viral replication in EECs and GFFs. Combined with our published study [39], we reasonably concluded that host lncRNAs and proteins work together to enhance the innate immune response and restrict viral replication in EECs during PPRV infection.

In this study, we confirmed that lncRNA IRF1-AS enhanced the phosphorylation of IRF3, promoted the production of IFN-β and ISGs and significantly inhibited viral infection. In the following study, we noticed that IRF1-AS was transcribed from the antisense strand in the opposite direction relative to IRF1. Another aspect of lncRNAs is that they play a regulatory role by interacting with their neighbouring protein-coding genes [61]. Next, we demonstrated a positive correlation between IRF1-AS and IRF1. IRF1 was the first IRF identified [62]. The function of IRF1 in innate immunity has recently received more attention. IRF1 is an extensively characterized ISG and a central regulator of the IFN response [45]. IRF1 can positively regulate the innate immune system to inhibit viral replication [63, 64]. One recent study showed that IRF1 binds to the promoter region of STAT1 to induce the transcription of ISGs, thus inhibiting hepatitis E virus (HEV) replication [65]. Here, we found that IRF1 positively regulated type I IFN production and ISG expression during PPRV infection. It is worth noticing that IRF1 appears to play a more important role than IRF3 and IRF7 in the induction of type III IFN [66], and IRF1 controls the induction of type III IFN by many pathogens [67, 68]. Therefore, we speculate that IRF1 is very likely to participate in the production of type III IFN, which plays another antiviral role in PPRV infection, and this possibility needs more study in the future.

IRF3 phosphorylation is a crucial step in the induction of IFNs [69, 70]. In our previous study, we demonstrated that PPRV infection can activate ISGs through IFN-independent and IRF3-dependent pathways [38]. Some studies have suggested many ways in which post-translational regulation impacts the IFN signalling pathway by regulating IRF3 [71–73]. Zhu’s finding also revealed that PPRV nucleocapsid protein inhibits beta interferon production by interacting with IRF3 to block its activation [37]. IRF1 and IRF3 are activated independently of each other [66]. However, one recent study showed that IRF1 interacts directly with IRF3 and augments the activation of IRF3 by blocking the interaction between IRF3 and protein phosphatase 2 A (PP2A) [47]. In our present study, we found that knockdown of IRF1 significantly inhibited the activation of IRF3. Additionally, our results indicated that PPRV infection can induce the interaction of IRF1 and IRF3 in nucleus. However, why the interaction of IRF1 and IRF3 can augment the innate immune response needs to be further investigated.

In summary, we examined the lncRNA profile changes in EECs in response to PPRV infection by deep sequencing. This study supports previous studies indicating the importance of the lncRNA landscape in the replication and pathogenesis of PPRV. We determined that IRF1-AS contributed to the production of type I IFN and ISGs by enhancing the phosphorylation of the key innate immune molecule IRF3 during PPRV infection, which could counteract the innate immunosuppression and suppress viral replication. Our findings provide a better understanding of host responses to PPRV infection and new directions for understanding the potential association between lncRNAs and PPRV pathogenesis.

## Supplementary Information

**Additional file 1.** ***Cis*** **model predicated target genes of DE lncRNAs.** 600 target genes for 162 DE lncRNAs were predicated by searching for protein coding genes within 100 kb of each DE lncRNA.

**Additional file 2.** ***RNAplex*** **software predicated target genes of DE lncRNAs.** *RNAplex* software was used to predict the complementary correlation of lncRNAs and mRNAs. It was found that 79 antisense lncRNAs had a complementary relationship with 284 mRNAs, which were considered one part of DE lncRNA targets.

**Additional file 3. A total of 852 target genes for 191 DE lncRNAs in the mock-infected and PPRV-infected groups were predicted.** The union of target genes predicated by *cis* and target genes predicated by *RNAplex* was seen as the target genes for all DE lncRNAs.

**Additional file 4. Target mRNAs related to immune system.** According to the GO annotation, 141 target mRNAs were classified as the BP term immune system.

## Data Availability

The datasets presented in this study can be found in online repositories. The data is deposited in the NCBI repository, accession number is PRJNA828346.
